# Pathogenesis, Symptomatology, and Transmission of SARS-CoV-2 through analysis of Viral Genomics and Structure

**Published:** 2021-02-01

**Authors:** Halie M. Rando, Adam L. MacLean, Alexandra J. Lee, Sandipan Ray, Vikas Bansal, Ashwin N. Skelly, Elizabeth Sell, John J. Dziak, Lamonica Shinholster, Lucy D’Agostino McGowan, Marouen Ben Guebila, Nils Wellhausen, Sergey Knyazev, Simina M. Boca, Stephen Capone, Yanjun Qi, YoSon Park, Yuchen Sun, David Mai, Christian Brueffer, James Brian Byrd, Jinhui Wang, Ronan Lordan, Ryan Velazquez, Gregory L Szeto, John P. Barton, Rishi Raj Goel, Serghei Mangul, Tiago Lubiana, COVID-19 Review Consortium, Anthony Gitter, Casey S. Greene

**Affiliations:** Department of Systems Pharmacology and Translational Therapeutics, University of Pennsylvania, Philadelphia, Pennsylvania, United States of America; Department of Biochemistry and Molecular Genetics, University of Colorado School of Medicine, Aurora, Colorado, United States of America; Center for Health AI, University of Colorado School of Medicine, Aurora, Colorado, United States of America · Funded by the Gordon and Betty Moore Foundation (GBMF 4552); Department of Biological Sciences, University of Southern California, Los Angeles, California, United States of America; Department of Systems Pharmacology and Translational Therapeutics, University of Pennsylvania, Philadelphia, Pennsylvania, United States of America · Funded by the Gordon and Betty Moore Foundation (GBMF 4552); Department of Biotechnology, Indian Institute of Technology Hyderabad, Kandi, Sangareddy 502285, Telangana, India; Biomedical Data Science and Machine Learning Group, German Center for Neurodegenerative Diseases, Tübingen 72076, Germany; Perelman School of Medicine, University of Pennsylvania, Philadelphia, Pennsylvania, United States of America; Institute for Immunology, University of Pennsylvania Perelman School of Medicine, Philadelphia, United States of America · Funded by NIH Medical Scientist Training Program T32 GM07170; Perelman School of Medicine, University of Pennsylvania, Philadelphia, Pennsylvania, United States of America; Edna Bennett Pierce Prevention Research Center, The Pennsylvania State University, University Park, PA, United States of America; Mercer University, Macon, GA, United States of America · Funded by the Center for Global Genomics and Health Equity at the University of Pennsylvania; Department of Mathematics and Statistics, Wake Forest University, Winston-Salem, North Carolina, United States of America; Department of Biostatistics, Harvard School of Public Health, Boston, Massachusetts, United States of America; Department of Systems Pharmacology and Translational Therapeutics, University of Pennsylvania, Philadelphia, Pennsylvania, United States of America; Georgia State University, Atlanta, GA, United States of America; Innovation Center for Biomedical Informatics, Georgetown University Medical Center, Washington, District of Columbia, United States of America; St. George’s University School of Medicine, St. George’s, Grenada; Department of Computer Science, University of Virginia, Charlottesville, VA, United States of America; Department of Systems Pharmacology and Translational Therapeutics, University of Pennsylvania, Philadelphia, Pennsylvania, United States of America · Funded by NHGRI R01 HG10067; Department of Computer Science, University of Virginia, Charlottesville, VA, United States of America; Department of Bioengineering, University of Pennsylvania, Philadelphia, PA, USA; Department of Clinical Sciences, Lund University, Lund, Sweden; University of Michigan School of Medicine, Ann Arbor, Michigan, United States of America · Funded by NIH K23HL128909; FastGrants; Perelman School of Medicine, University of Pennsylvania, Philadelphia, Pennsylvania, United States of America; Institute for Translational Medicine and Therapeutics, Perelman School of Medicine, University of Pennsylvania, Philadelphia, PA 19104-5158, USA; Azimuth1, McLean, VA; Allen Institute for Immunology, Seattle, WA, United States of America; Department of Physics and Astronomy, University of California-Riverside, Riverside, California, United States of America; Institute for Immunology, University of Pennsylvania, Philadelphia, PA, United States of America; Department of Clinical Pharmacy, School of Pharmacy, University of Southern California, Los Angeles, CA, United States of America; Department of Clinical and Toxicological Analyses, School of Pharmaceutical Sciences, University of São Paulo, São Paulo, Brazil; Department of Biostatistics and Medical Informatics, University of Wisconsin-Madison, Madison, Wisconsin, United States of America; Morgridge Institute for Research, Madison, Wisconsin, United States of America · Funded by John W. and Jeanne M. Rowe Center for Research in Virology; Department of Systems Pharmacology and Translational Therapeutics, University of Pennsylvania, Philadelphia, Pennsylvania, United States of America; Childhood Cancer Data Lab, Alex’s Lemonade Stand Foundation, Philadelphia, Pennsylvania, United States of America; Department of Biochemistry and Molecular Genetics, University of Colorado School of Medicine, Aurora, Colorado, United States of America; Center for Health AI, University of Colorado School of Medicine, Aurora, Colorado, United States of America · Funded by the Gordon and Betty Moore Foundation (GBMF 4552); the National Human Genome Research Institute (R01 HG010067)

## Abstract

The novel coronavirus SARS-CoV-2, which emerged in late 2019, has since spread around the world infecting tens of millions of people with coronavirus disease 2019 (COVID-19). While this viral species was unknown prior to January 2020, its similarity to other coronaviruses that infect humans has allowed for rapid insight into the mechanisms that it uses to infect human hosts, as well as the ways in which the human immune system can respond. Here, we contextualize SARS-CoV-2 among other coronaviruses and identify what is known and what can be inferred about its behavior once inside a human host. Because the genomic content of coronaviruses, which specifies the virus’s structure, is highly conserved, early genomic analysis provided a significant head start in predicting viral pathogenesis. The pathogenesis of the virus offers insights into symptomatology, transmission, and individual susceptibility. Additionally, prior research into interactions between the human immune system and coronaviruses has identified how these viruses can evade the immune system’s protective mechanisms. We also explore systems-level research into the regulatory and proteomic effects of SARS-CoV-2 infection and the immune response. Understanding the structure and behavior of the virus serves to contextualize the many facets of the COVID-19 pandemic and can influence efforts to control the virus and treat the disease.

## Introduction

The current coronavirus disease 2019 (COVID-19) pandemic, caused by the *Severe acute respiratory syndrome-related coronavirus 2* (SARS-CoV-2) virus, represents an acute global health crisis. Symptoms of the disease can range from mild to severe or fatal [1] and can affect a variety of organs and systems [2]. Outcomes of infection can include acute respiratory distress (ARDS) and acute lung injury, as well as damage to other organ systems [2,3]. Understanding the progression of the disease, including these diverse symptoms, depends on understanding how the virus interacts with the host. Additionally, the fundamental biology of the virus can provide insights into how it is transmitted among people, which can, in turn, inform efforts to control its spread. As a result, a thorough understanding of the pathogenesis of SARS-CoV-2 is a critical foundation on which to build an understanding of COVID-19 and the pandemic as a whole.

The rapid identification and release of the genomic sequence of the virus in January 2020 [4] provided early insight into the virus in a comparative genomic context. The viral genomic sequence clusters with known coronaviruses (order *Nidovirales*, family *Coronaviridae*, subfamily *Orthocoronavirinae*). Phylogenetic analysis of the coronaviruses reveals four major subclades, each corresponding to a genus: the alpha, beta, gamma, and delta coronaviruses. Among them, alpha- and betacoronaviruses infect mammalian species, gammacoronaviruses infect avian species, and deltacoronaviruses infect both mammalian and avian species [5]. The novel virus now known as SARS-CoV-2 was identified as a betacoronavirus belonging to the B lineage based on phylogenetic analysis of a polymerase chain reaction (PCR) amplicon fragment from five patients along with the full genomic sequence [6]. This lineage also includes the *Severe acute respiratory syndrome-related coronavirus* (SARS-CoV-1) that caused the 2002–2003 outbreak of Severe Acute Respiratory Syndrome (SARS) in humans [6]. Because viral structure and mechanisms of pathogenicity are highly conserved within the order, this phylogenetic analysis provided a basis for forming hypotheses about how the virus interacts with hosts, including which tissues, organs, and systems would be most susceptible to SARS-CoV-2 infection. Coronaviruses that infect humans (HCoV) are not common, but prior research into other HCoV such as SARS-CoV-1 and *Middle East respiratory syndrome-related coronavirus* (MERS-CoV), as well as other viruses infecting humans such as a variety of influenza species, established a strong foundation that accelerated the pace of SARS-CoV-2 research.

**Figure 1:**
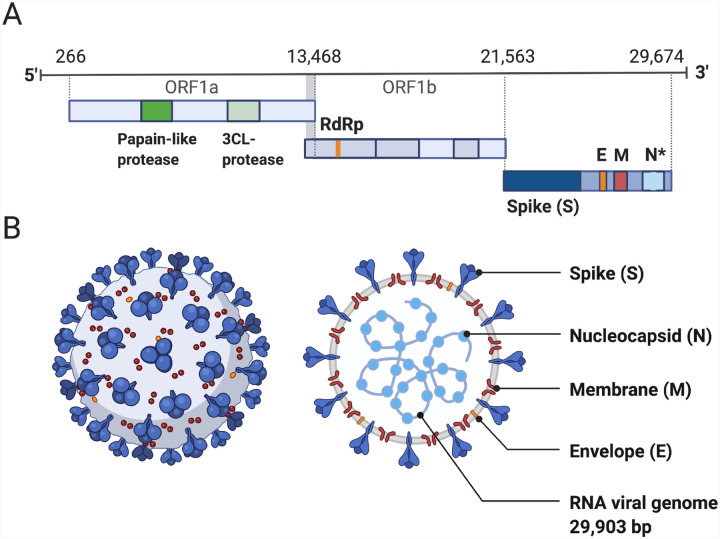
Structure of SARS-CoV-2 capsid and genome. A) The genomic structure of coronaviruses is highly conserved and includes three main regions. Open reading frames (ORF) 1a and 1b contain two polyproteins that encode the non-structural proteins (nsp). The nsp include proteases such as RNA-dependent RNA Polymerase (RdRp). The last third of the genome encodes structural proteins, including the spike (S), envelope (E), membrane (M) and nucleocapsid (N) proteins. Accessory genes can also be interspersed throughout the genome [11]. B) The physical structure of the coronavirus virion, including the components determined by the conserved structural proteins S, E, M and N.

Coronaviruses are large viruses that can be identified by their distinctive “crown-like” shape (Figure 1). Their spherical virions are made from lipid envelopes ranging from 100 to 160 nanometers in which peplomers (protruding structures) of two to three spike (S) glycoproteins are anchored, creating the crown [7,8]. These spikes, which are critical to both viral pathogenesis and to the response by the host immune response, have been visualized using cryo-electron microscopy [9]. Because they induce the human immune response, they are also the target of many proposed therapeutic agents. Viral pathogenesis is typically broken down into three major components: entry, replication, and spread [10]. However, in order to draw a more complete picture of pathogenesis, it is also necessary to examine how infection manifests clinically, identify systems-level interactions between the virus and the human body, and consider the possible effects of variation or evolutionary change on pathogenesis and virulence. Thus, clinical medicine and traditional biology are both important pieces of the puzzle of SARS-CoV-2 presentation and pathogenesis.

## Coronavirus Structure and Pathogenesis

### Structure of Coronaviruses

Genome structure is highly conserved among coronaviruses, meaning that the relationship between the SARS-CoV-2 genome and its pathogenesis can be inferred from prior research in related viral species. The genomes of viruses in the *Nidovirales* order share several fundamental characteristics. They are non-segmented, which means the viral genome is contained in a single capsid, and are enveloped, which means that the genome and capsid are encased by a lipid bilayer. Coronaviruses have large positive-sense RNA (ssRNA+) genomes ranging from 27 to 32 kilobases in length [11,12]. The SARS-CoV-2 genome lies in the middle of this range at 29,903 bp [12]. Genome organization is highly conserved within the order [11]. There are three major genomic regions: one containing the replicase gene, one containing the genes encoding structural proteins, and interspersed accessory genes [11] (Figure 1). The replicase gene comprises about two-thirds of the genome and consists of two open reading frames that are translated with ribosomal frameshifting [11]. This polypeptide is then translated into 16 non-structural proteins (nsp), except in gammacoronaviruses where nsp1 is absent, that form replication machinery used to synthesize viral RNA [13]. The remaining third of the genome encodes structural proteins, including the spike, membrane, envelope, and nucleocapsid proteins. Additional accessory genes are sometimes present between these two regions, depending on the species or strain. Much attention has been focused on the S protein, which is a critical structure involved in cell entry.

### Pathogenic Mechanisms of Coronaviruses

While, like most viruses, it is possible that SARS-CoV-1 and SARS-CoV-2 can enter cells through endocytosis, coronaviruses are also able to target cells for entry through fusion with the plasma membrane [14,15]. This process is conserved among coronaviruses and is closely associated with the content of their genomes. Cell entry proceeds in three steps: binding, cleavage, and fusion. First, the viral spike protein binds to a host cell via a recognized receptor or entry point. Coronaviruses can bind to a range of host receptors [16,17], with binding conserved only at the genus level [5]. Viruses in the betacoronavirus genus, to which SARS-CoV-2 belongs, are known to bind to the CEACAM1 protein, 5-N-acetyl-9-O-acetyl neuraminic acid, and to the angiotensin-converting enzyme 2 (ACE2) [16]. SARS-CoV-2 has a high affinity for human ACE2, which is expressed in the vascular epithelium, other epithelial cells, and cardiovascular and renal tissues [18,19], as well as many others [20]. The binding process is guided by the molecular structure of the spike protein, which is structured in three segments: an ectodomain, a transmembrane anchor, and an intracellular tail [21]. The ectodomain forms the crown-like structures on the viral membrane and contains two subdomains known as the S1 and S2 subunits [22]. The S1 (N-terminal) domain forms the head of the crown and contains the receptor binding motif, and the S2 (C-terminal) domain forms the stalk that supports the head [22]. The S1 subunit guides the binding of the virus to the host cell, and the S2 subunit guides the fusion process [21].

After the binding of the S1 subunit to an entry point, the spike protein is often cleaved at the S1-S2 boundary by a host protease [23,24,25]. Similar to SARS-CoV-1, SARS-CoV-2 exhibits redundancy in which host proteases can cleave the S protein [26]. Specifically, both transmembrane protease serine protease-2 (TMPRSS2) and cathepsins B/L have been shown to mediate SARS-CoV-2 S protein proteolytic priming, and small molecule inhibition of these enzymes fully inhibited viral entry *in vitro* [26,27]. Proteolytic priming prepares the S protein for fusion [24,25]. The two subunits remain bound by van der Waals forces, with the S1 subunit stabilizing the S2 subunit during the membrane fusion process [23]. Electron microscopy suggests that in some coronaviruses, including SARS-CoV-1 and MERS-CoV, a six-helix bundle separates the two subunits in the postfusion conformation, and the unusual length of this bundle facilitates membrane fusion through the release of additional energy [5]. Cleavage at a second site within S2 by these same proteases activates *S* for fusion by inducing conformational changes [23]. The viral membrane can then fuse with the endosomal membrane to release the viral genome into the host cytoplasm. Once the virus enters a host cell, the replicase gene is translated and assembled into the viral replicase complex. This complex then synthesizes the double-stranded RNA (dsRNA) genome from the genomic ssRNA(+). The dsRNA genome is transcribed and replicated to create viral mRNAs and new ssRNA(+) genomes [11,28]. From there, the virus can spread into other cells. In this way, the genome of SARS-CoV-2 provides insight into the pathogenic behavior of the virus.

### Immune Evasion Strategies

Research in other HCoV provides some indication of how SARS-CoV-2 infection proceeds in spite of the human immune response. By infecting the epithelium, viruses such as SARS-CoV-1 are known to bypass the physical barriers, such as skin and mucus, that comprise the immune system’s first line of defense [29]. Once the virus infiltrates host cells, it is adept at evading detection. CD163+ and CD68+ macrophage cells are especially crucial for the establishment of SARS-CoV-1 in the body [29]. These cells most likely serve as viral reservoirs that help shield SARS-CoV-1 from the innate immune response. According to a study on the viral dissemination of SARS-CoV-1 in Chinese macaques, viral RNA could be detected in some monocytes throughout the process of differentiation into dendritic cells [29]. This lack of active viral replication allows SARS-CoV-1 to escape the innate immune response because reduced levels of detectable viral RNA allow the virus to avoid both natural killer cells and Toll-like receptors [29]. Even during replication, SARS-CoV-1 is able to mask its dsRNA genome from detection by the immune system. Although dsRNA is a pathogen-associated molecular pattern that would typically initiate a response from the innate immune system [30], *in vitro* analysis of nidoviruses including SARS-CoV-1 suggests that these viruses can induce the development of double-membrane vesicles that protect the dsRNA signature from being detected by the host immune system [31]. This protective envelope can therefore insulate these coronaviruses from the innate immune system’s detection mechanism [32].

HCoVs are also known to interfere with the host immune response, rather than just evade it. For example, the virulence of SARS-CoV-2 is increased by nsp1, which can suppress host gene expression by stalling mRNA translation and inducing endonucleolytic cleavage and mRNA degradation [33]. SARS-CoV-1 also evades the immune response by interfering with type I IFN induction signaling, which is a mechanism that leads to cellular resistance to viral infections. SARS-CoV-1 employs methods such as ubiquitination and degradation of RNA sensor adaptor molecules MAVS and TRAF3/6 [34]. Also, MERS-CoV downregulates antigen presentation via MHC class I and MHC class II, which leads to a reduction in T cell activation [34]. These evasion mechanisms, in turn, can facilitate systemic infection. Coronaviruses such as SARS-CoV-1 are also able to evade the humoral immune response through other mechanisms, such as inhibiting certain cytokine pathways or down-regulating antigen presentation by the cells [31].

### Host Cell Susceptibility

ACE2 and TMPRSS2 have been identified as the primary entry portal and as a critical protease, respectively, in facilitating the entry of SARS-CoV-1 and SARS-CoV-2 into a target cell [9,26,35,36,37]. This finding has led to a hypothesized role for ACE2 and TMPRSS2 expression in determining which cells, tissues, and organs are most likely to be infected by SARS-CoV-2. ACE2 is expressed in numerous organs, such as the heart, kidney, and intestine, but it is most prominently expressed in alveolar epithelial cells; this pattern of expression is expected to contribute to the virus’ association with lung pathology [18,38,39] as well as that of SARS [40]. Clinical investigations of COVID-19 patients have detected SARS-CoV-2 transcripts in bronchoalveolar lavage fluid (BALF) (93% of specimens), sputum (72%), nasal swabs (63%), fibrobronchoscopy brush biopsies (46%), pharyngeal swabs (32%), feces (29%), and blood (1%) [41]. Two studies reported that SARS-CoV-2 could not be detected in urine specimens [41,42]; however, a third study identified four urine samples (out of 58) that were positive for SARS-CoV-2 nucleic acids [43]. Although respiratory failure remains the leading cause of death for COVID-19 patients [44], SARS-CoV-2 infection can damage many other organ systems including the heart [45], kidneys [46,47], liver [48], and gastrointestinal tract [49,50]. As it becomes clear that SARS-CoV-2 infection can damage multiple organs, the scientific community is pursuing multiple avenues of investigation in order to build a consensus about how the virus affects the human body.

## Clinical Presentation of COVID-19

SARS-CoV-2 pathogenesis is closely linked with the clinical presentation of the COVID-19 disease. Reports have described diverse symptom profiles associated with COVID-19, with a great deal of variability both within and between institutions and regions. A large study from Wuhan, China conducted early in the pandemic identified fever and cough as the two most common symptoms that patients reported at hospital admission [51], while a retrospective study in China described the clinical presentations of patients infected with SARS-CoV-2 as including lower respiratory tract infection with fever, dry cough, and dyspnea (shortness of breath) [52]. This study [52] noted that upper respiratory tract symptoms were less common, suggesting that the virus preferentially targets cells located in the lower respiratory tract. However, data from the New York City region [53,54] showed variable rates of fever as a presenting symptom, suggesting that symptoms may not be consistent across individuals. For example, even within New York City, one study [53] identified low oxygen saturation (<90% without the use of supplemental oxygen or ventilation support) in 20.4% of patients upon presentation, with fever being present in 30.7%, while another study [51] reported cough (79.4%), fever (77.1%), and dyspnea (56.5%) as the as the most common presenting symptoms; both of these studies considered only hospitalized patients. A later study reported radiographic findings such as ground-glass opacity and bilateral patchy shadowing in the lungs of many hospitalized patients, with most COVID-19 patients having lymphocytopenia, or low levels of lymphocytes (a type of white blood cell) [51]. Patients may also experience loss of smell, myalgias (muscle aches), fatigue, or headache. Gastrointestinal symptoms can also present [55], and the CDC includes nausea and vomiting, as well congestion and runny nose, on its list of symptoms consistent with COVID-19 [1]. A preprint using data from an app-based survey of 500,000 individuals in the US found that among those tested for SARS-CoV-2, a loss of taste or smell, fever, and a cough were significant predictors of a positive test result [56]. It is important to note that in this study, the predictive value of symptoms may be underestimated if they are not specific to COVID-19. This underestimation could occur because the outcome measured was a positive, as opposed to a negative, COVID-19 test result, meaning an association would be more easily identified for symptoms that were primarily or exclusively found with COVID-19. At the time the surveys were conducted, due to limits in US testing infrastructure, respondents typically needed to have some symptoms known to be specific to COVID-19 in order to qualify for testing. Widespread testing of asymptomatic individuals may therefore provide additional insight into the range of symptoms associated with COVID-19.

Consistent with the wide range of symptoms observed and the pathogenic mechanisms described above, COVID-19 can affect diverse body systems in addition to causing respiratory problems [57]. For example, COVID-19 can lead to acute kidney injury, especially in patients with severe respiratory symptoms or certain preexisting conditions [58]. It can also cause neurological complications [59,60], potentially including stroke, seizures or meningitis [61,62]. In fact, autopsy samples suggest that SARS-CoV-2 may be able to enter the central nervous system via the neural–mucosal interface [63]. COVID-19 has also been associated with an increased incidence of large vessel stroke, particularly in patients under the age of 40 [64], and other thrombotic events including pulmonary embolism and deep vein thrombosis [65]. The mechanism behind these complications has been suggested to be related to coagulopathy, with reports indicating the presence of antiphospholipid antibodies [66] and elevated levels of d-dimer and fibrinogen degradation products in deceased patients [67]. Other viral infections have been associated with coagulation defects and changes to the coagulation cascade; notably, SARS was also found to lead to disseminated intravascular coagulation and was associated with both pulmonary embolism and deep vein thrombosis [68]. The mechanism behind these insults has been suggested to be related to inflammation-induced increases in the von Willebrand factor clotting protein, leading to a pro-coagulative state [68]. Abnormal clotting (thromboinflammation or coagulopathy) has been increasingly discussed recently as a possible key mechanism in many cases of severe COVID-19, and may be associated with the high d-dimer levels often observed in severe cases [69,70,71]. This excessive clotting in lung capillaries has been suggested to be related to a dysregulated activation of the complement system, part of the innate immune system [72,73].

### Cytokine Release Syndrome

Symptoms of a disease can be caused by a pathogen, but they can also be caused by the immune system’s reaction to the pathogen. A dysregulated immune response can cause significant damage to the host [74,75,76]. The inflammatory response has received particular attention for its role in both a healthy response to infection and a pathogenic one. Inflammation is one of the most visible components of the immune response, as it is responsible for the hallmarks of injury, such as pain, heat, and swelling [77]. In response to injury or to signaling by pattern recognition receptors indicating the detection of a molecular pattern associated with a pathogen or foreign body, the immune system stimulates leukocytes that travel to the site of the threat, where they then produce cytokines [77]. Cytokines are a diverse group of small proteins that play an important role in intercellular signaling [78]. Cytokines can be both pro- and anti-inflammatory, which means they can either stimulate or inhibit the production of additional cytokines [78,79]. Some notable pro-inflammatory cytokines include the interleukins IL-1β and IL-6 and tumor necrosis factor α (TNF-α) [79]. Anti-inflammatory cytokines play an immunoregulatory role complementary to the cascading effect of pro-inflammatory cytokines [78,79]. A number of interleukins and interferons play anti-inflammatory roles, and receptors or receptor antagonists for inflammatory cytokines are also important for regulating inflammation [79]. IL-10 is an anti-inflammatory cytokine of particular note because it regulates the expression of TNF-α, IL-1, and IL-6 [79]. When the pro- and anti-inflammatory responses are both commensurate with the threat posed, the immune system drives a shift back to homeostasis [80]. However, when the responses are disproportionate, the cytokine response can become dysregulated. Too low of an inflammatory response will not eliminate the immune threat [80]. In contrast, if the response is dysregulated towards excessive pro-inflammatory cytokine activity, inflammation can cascade [81] and cause cell damage, among other problems [77]. Elevated levels of inflammation over the long-term are associated with many chronic health conditions, including type 2 diabetes, dementia and Alzheimer’s, and arthritis [82]. On a shorter timescale, dysregulated systemic inflammation can cause sepsis, which can lead to multi-organ failure and death [78,83].

Cytokines have been investigated for their role in the immune response to lung infections long before the COVID-19 pandemic. Dysregulation of the inflammatory response, including elevated levels of pro-inflammatory cytokines, is found in patients with ARDS, which is a severe condition that can arise from pneumonia, SARS, and COVID-19 [81]. One study of patients with and at risk for ARDS, specifically those who were intubated for medical ventilation, found that shortly after the onset of ARDS, anti-inflammatory cytokine concentration in BALF increased relative to the concentration of pro-inflammatory cytokines [84]. The results suggest that an increase in pro-inflammatory cytokines such as IL-6 may signal the onset of ARDS, but recovery depends on an increased anti-inflammatory response [84]. However, patients with severe ARDS were excluded from this study. Acute phase response to an infection can also cause damage to the capillary endothelium, allowing leaks that disrupt the balance between pro-inflammatory cytokines and their regulators [84]. Hyperactivity of the pro-inflammatory response due to lung infection is commonly associated with acute lung injury and more rarely with the more severe manifestation, ARDS [78]. The heightened inflammatory response in the lungs can also serve as a source for systemic inflammation, or sepsis, and potentially multi-organ failure [78]. The shift from local to systemic inflammation is a phenomenon often referred to broadly as a cytokine storm [78] or, more precisely, as cytokine release syndrome [85]. Sepsis is a known possible complication of pneumonia, and in an analysis of over 1,400 US pneumonia patients, IL-6, tumor necrosis factor (TNF), and IL-10 were found to be elevated at intake in patients who developed severe sepsis and/or ultimately deceased [86]. However, unlike the study analyzing pro- and anti-inflammatory cytokines in ARDS patients [84], this study reported that unbalanced pro-/anti-inflammatory cytokine profiles were rare. This discrepancy could be related to the fact that the sepsis study measured only three cytokines. Regardless of variation in the anti-inflammatory response, prior work has therefore made it clear that pulmonary infection and injury are associated with systemic inflammation and with sepsis. While IL-6 is a biomarker sometimes used to assess cytokine storm activity in sepsis [78], the relationship between cytokine profiles and the risks associated with sepsis may be more complex. In fact, although IL-6 has traditionally been considered pro-inflammatory, its pleiotropic effects via both classical and trans-signaling allow it to play an integral role in both the inflammatory and anti-inflammatory responses [87], leading it to be associated with both healthy and pathological responses to viral threat [88].

The inflammatory response was identified early on as a potential driver of COVID-19 outcomes due to existing research in SARS and emerging research in COVID-19. In addition to the known role of cytokines in ARDS and lung infection more broadly, immunohistological analysis at autopsy of patients deceased from SARS revealed that ACE2-expressing cells that were infected by SARS-CoV-1 showed elevated expression of IL-6, IL-1β, and TNF-α [89]. Similarly, the introduction of the S protein from SARS-CoV-1 to mouse macrophages was found to increase production of IL-6 and TNF-α [90]. For SARS-CoV-2 infection leading to COVID-19, early reports described a cytokine storm syndrome-like response in patients with particularly severe infections [38,91,92]. Among patients hospitalized with COVID-19 in Wuhan, China, 112 out of 191 (59%) developed sepsis, including all 54 of the non-survivors [52]. However, the argument has been made that while the cytokine levels observed in COVID-19 patients fall outside of the normal range, they are not as high as typically found in patients with ARDS [93]. Regardless, inflammation has received significant interest both in regards to the pathology of COVID-19 as well as potential avenues for treatment, as the relationship between the cytokine storm and the pathophysiology of COVID-19 has led to the suggestion that a number of immunomodulatory pharmaceutical interventions could hold therapeutic value for the treatment of COVID-19 [94].

### Pediatric Presentation

The presentation of COVID-19 infection can vary greatly among pediatric patients and, in some cases, manifests in distinct ways from COVID-19 in adults. Evidence suggests that while children and adolescents tend to have mostly asymptomatic infections, those that are symptomatic typically exhibit a mild illness [95,96,97,98]. One review examined symptoms reported in 17 studies of children infected with COVID-19 during the early months of the COVID-19 epidemic in China and one study from Singapore [99]. In the more than a thousand cases described, the most common reports were for mild symptoms such as fever, dry cough, fatigue, nasal congestion and/or runny nose, while three children were reported to be asymptomatic. Severe lower respiratory infection was described in only one of the pediatric cases reviewed. Gastrointestinal symptoms such as vomiting or diarrhea were occasionally reported. Radiologic findings were not always reported in the case studies reviewed, but when they were mentioned they included bronchial thickening, ground-glass opacities, and/or inflammatory lesions [99]. Neurological symptoms have also been reported [100].

These analyses indicate that most pediatric cases of COVID-19 are not severe. Indeed, it is estimated that less than 1% of pediatric cases result in critical illness [97,101]. However, serious complications and, in rare cases, deaths have occurred [102]. Of particular interest, children have occasionally experienced a serious inflammatory syndrome, multisystem inflammatory syndrome in children (MIS-C), following COVID-19 infection. This syndrome is similar in some respects to Kawasaki disease, including Kawasaki disease shock syndrome [103,104,105] and is thought to be a distinct clinical manifestation of SARS-CoV-2 due to its distinct cytokine profile and the presence of burr cells in peripheral blood smears [106,107]. MIS-C has been associated with heart failure in some cases [108]. One case study [109] described an adult who appeared to show symptoms similar to MIS-C after exposure to COVID-19, but cautioned against broad conclusions; a second possible adult case has also been reported [110]. The presentation of SARS-CoV-2 infection is therefore likely to be largely distinct between adult and pediatric populations.

## Systems-Level Effects

Systems biology provides a cross-disciplinary analytical paradigm through which the host response to an infection can be analyzed. This field integrates the “omics” fields (genomics, transcriptomics, proteomics, metabolomics, etc.) using bioinformatics and other computational approaches. Over the last decade, systems biology approaches have been used widely to study the pathogenesis of diverse types of life-threatening acute and chronic infectious diseases [111]. Omics-based studies have also provided meaningful information regarding host immune responses and surrogate protein markers in several viral, bacterial and protozoan infections [112]. Though the complex pathogenesis and clinical manifestations of SARS-CoV-2 infection are not yet fully understood, omics technologies offer the opportunity for discovery-driven analysis of biological changes associated with SARS-CoV-2 infection. For example, previous studies suggest that infection by coronaviruses, such as SARS-CoV-1 and MERS-CoV, as well as other viruses, is associated with the upregulation of ACE2. In several preliminary assays and an analysis of microarray data, ACE2 expression was reported to be significantly upregulated following infection of human embryonic kidney cells and human airway epithelial cells [38]. This study also reported that direct stimulation with inflammatory cytokines such as type I interferons (e.g., IFNβ) resulted in the upregulation of ACE2 in human bronchial epithelial cells, with treated groups showing four-fold higher ACE2 expression than control groups at 18 hours post-treatment [38]. While it is still unclear whether SARS-CoV-2 facilitates the positive regulation of its own transmission between host cells, the host immune response itself likely plays a key role in mediating infection-associated pathologies. For this reason, the application of omics technologies to the process of characterizing the host response is expected to provide novel insights into how hosts respond to SARS-CoV-2 infection and how these changes might influence COVID-19 outcomes.

### Transcriptomics

In addition to the study described above [38], two other studies have profiled expression following SARS-CoV-2 infection using human cell lines. The first study [113] compared transcriptional responses to SARS-CoV-2 and to other respiratory viruses, including MERS-CoV, SARS-CoV, *Human parainfluenza virus 3*, *Respiratory syncytial virus*, and *Influenza A virus*. The responses of three human cell lines were analyzed: A549 (adenocarcinomic human alveolar basal epithelial cells), Calu-3 (human airway epithelial cells derived from human bronchial submucosal glands), and MRC-5 (human fetal lung fibroblast cells). As the viral entry portal ACE2 has low expression in A549 cells, these cells were supplemented with adenovirus-based vectors expressing either mCherry (a fluorescent protein used as a control) or ACE2 (A549-ACE2). The authors also measured host transcriptional responses to SARS-CoV-2 in primary normal human bronchial epithelial cells (HBEC or NHBE cells), nasal washes from an animal model (ferret), and lung samples from two COVID-19 patients. The transcriptional response differed between the COVID-19 infected cells and the cells infected by other viruses, with changes in differential expression specific to each infection type. In the hosts where SARS-CoV-2 was able to replicate efficiently, differential expression analysis revealed that the transcriptional response was significantly different from the response to all of the other viruses tested. A unique pro-inflammatory cytokine signature associated with SARS-CoV-2 was present in cells exposed to both high and low doses of the virus, with the cytokines IL-6 and IL1RA uniquely elevated in response to SARS-CoV-2 relative to other viruses. However, the A549-ACE2 cells showed significant IFN-I or IFN-III expression when exposed to high, but not low, doses of SARS-CoV-2. This finding suggests that IFN induction is dependent on the extent of exposure. Similarly, in cells from the NHBE line, ferrets, and COVID-19 patients, chemokine signaling was significantly enriched, but there was no significant induction of IFN-I or IFN-III. Together, these results suggest that SARS-CoV-2 induces a limited antiviral state with low IFN-I or IFN-III expression and a moderate IFN-stimulated gene response, in contrast to other viruses. Other respiratory viruses have been found to encode antagonists to the IFN response. The analysis of SARS-CoV-2 suggested that this transcriptional state was specific to cells expressing ACE2, as it was not observed in cells lacking expression of this protein except with ACE2 supplementation and at very high (10-fold increase) level of SARS-CoV-2 exposure. This hypothesis was further supported by a recent study [114] that showed that the SARS-CoV-2 *ORF3b* gene suppresses IFNB1 promoter activity (IFN-I induction) more efficiently than the SARS-CoV-1 *ORF3b* gene. Taken together, these findings suggest that a unique cytokine profile is associated with the response to the SARS-CoV-2 virus, and that this response differs depending on the extent of exposure.

Another study [115] analyzed dynamic transcriptional responses to SARS-CoV-2 and SARS-CoV-1. They characterized the response of three human cell lines, H1299 (human non-small cell lung carcinoma cell line), Calu-3, and Caco-2 (human epithelial colorectal adenocarcinoma cell line), at 4 to 36 hours post infection. Using poly(A) bulk RNA-seq, the authors found negligible susceptibility of H1299 cells (< 0.08 viral read percentage of total reads) compared to Caco-2 and Calu-3 cells (>10% of viral reads). This finding suggests that the risk of infection varies among cell types, and that cell type could influence which hosts are more or less susceptible. Based on visual inspection of microscopy images alongside transcriptional profiling, the authors also showed distinct responses among the host cell lines evaluated. In contrast to Caco-2, Calu-3 cells infected with SARS-CoV-2 showed signs of impaired growth and cell death at 24 hours post infection, as well as moderate IFN induction with a strong up-regulation of IFN-stimulated genes. Interestingly, the results were similar to those reported in Calu-3 cells exposed to much higher levels of SARS-CoV-2 [113], as described above. This finding suggests that IFN induction in Calu-3 cells is not dependent on the level of exposure, in contrast to A549- ACE2 cells. The discrepancy could be explained by the observations that Calu-3 cells are highly susceptible to SARS-CoV-2 and show rapid viral replication [27], whereas A549 cells are incompatible with SARS-CoV-2 infection [116]. This discrepancy raises the concern that *in vitro* models may vary in their similarity to the human response, underscoring the importance of follow-up studies in additional models.

### Proteomics

One early proteomics study investigated changes associated with *in vitro* SARS-CoV-2 infection using Caco-2 cells [117]. This study reported that SARS-CoV-2 induced alterations in multiple vital physiological pathways, including translation, splicing, carbon metabolism and nucleic acid metabolism in the host cells. Another area of interest is whether SARS-CoV-2 is likely to induce similar changes to other HCoV. For example, because of the high level of sequence homology between SARS-CoV-2 and SARS-CoV-1, it has been hypothesized that sera from convalescent SARS-CoV-1 patients might show some efficacy in cross-neutralizing SARS-CoV-2-S-driven entry [26]. However, despite the high level of sequence homology, certain protein structures might be immunologically distinct, which would be likely to prohibit effective cross-neutralization across different SARS species [118]. Consequently, proteomic analyses of SARS-CoV-1 might also provide some essential information regarding the new pathogen [119,120].

Considering the paucity of omics-level big data sets for SARS-CoV-2 currently available, existing data hubs that contain information for other coronaviruses such as UniProt [121], NCBI Genome Database [122], The Immune Epitope Database and Analysis Resource [123], and The Virus Pathogen Resource [124] will serve as useful resources for comparative bioinformatics research of SARS-CoV-2. Using such databases, the systems-level reconstruction of protein-protein interaction networks will enable the generation of hypotheses about the mechanism of action of SARS-CoV-2 and suggest potential drug targets. In an initial study [125], 26 of the 29 SARS-CoV-2 proteins were cloned and expressed in HEK293T kidney cells, allowing for the identification of 332 high-confidence human proteins interacting with them. Notably, this study suggested that SARS-CoV-2 interacts with innate immunity pathways. Ranking pathogens by the similarity between their interactomes and that of SARS-CoV-2 suggested *West Nile virus*, *Mycobacterium tuberculosis*, and *human papillomavirus* infections as the top three hits. Therefore, given the lung symptoms associated with COVID-19, the *Mycobacterium tuberculosis* host-pathogen interactome in particular might provide new insights to the mechanism of SARS-CoV-2 infection. Additionally, it was suggested that the envelope protein, E, could disrupt host bromodomain-containing proteins, i.e., BRD2 and BRD4, that bind to histones, and the spike protein could likely intervene in viral fusion by modulating the GOLGA7-ZDHHC5 acyl-transferase complex to increase palmitoylation, which is a post-translational modification that affects how proteins interact with membranes [126].

Another study [127] used patient-derived peripheral blood mononuclear cells to identify 251 host proteins targeted by SARS-CoV-2. This study also reported that more than 200 host proteins were disrupted following infection. In particular, a network analysis showed that nsp9 and nsp10 interacted with NF-Kappa-B-Repressing Factor, which encodes a transcriptional repressor that mediates repression of genes responsive to Nuclear Factor kappa-light-chain-enhancer of activated B-cells. These genes are important to pro-, and potentially also anti-, inflammatory signaling [128]. This finding could explain the exacerbation of the immune response that shapes the pathology and the high cytokine levels characteristic of COVID-19, possibly due to the chemotaxis of neutrophils mediated by IL-8 and IL-6. Finally, it was suggested [129] that the E protein of both SARS-CoV-1 and SARS-CoV-2 has a conserved Bcl-2 Homology 3-like motif, which could inhibit anti-apoptosis proteins, e.g., BCL2, and trigger the apoptosis of T cells. Several compounds are known to disrupt the host-pathogen protein interactome, largely through the inhibition of host proteins. Therefore, this research identifies candidate targets for intervention and suggests that drugs modulating protein-level interactions between virus and host could be relevant to treating COVID-19. By revealing which genes are perturbed during SARS-CoV-2 infection, proteomics-based analyses can thus provide novel insights into host-virus interaction and serve to generate new avenues of investigation for therapeutics.

## Viral Virulence

Like that of SARS-CoV-1, the entry of SARS-CoV-2 into host cells is mediated by interactions between the viral spike glycoprotein, S, and human ACE2 (hACE2) [23,26,130,131,132,133,134,135]. Differences in how the S proteins of the two viruses interact with hACE2 could partially account for the increased transmissibility of SARS-CoV-2. Recent studies have reported conflicting binding constants for the S-hACE2 interaction, though they have agreed that the SARS-CoV-2 S protein binds with equal, if not greater, affinity than the SARS-CoV-1 S protein does [9,23,133]. The C-terminal domain of the SARS-CoV-2 S protein in particular was identified as the key region of the virus that interacts with hACE2, and the crystal structure of the C-terminal domain of the SARS-CoV-2 S protein in complex with hACE2 reveals stronger interaction and a higher affinity for receptor binding than that of SARS-CoV-1 [134]. Among the 14 key binding residues identified in the SARS-CoV-1 S protein, eight are conserved in SARS-CoV-2, and the remaining six are semi-conservatively substituted, potentially explaining variation in binding affinity [23,133]. Recent crystal structures have shown that the receptor binding domain (RBD) of the SARS-CoV-2 S protein, like that of other coronaviruses, undergoes stochastic hinge-like movement that flips it from a “closed” conformation, in which key binding residues are hidden at the interface between protomers, to an “open” one [9,23]. Because the RBD plays such a critical role in viral entry, blocking its interaction with ACE2 could represent a promising therapeutic approach. Nevertheless, despite the high structural homology between the SARS-CoV-2 RBD and that of SARS-CoV-1, monoclonal antibodies targeting SARS-CoV-1 RBD failed to bind to SARS-CoV-2-RBD [9]. However, in early research, sera from convalescent SARS patients were found to inhibit SARS-CoV-2 viral entry *in vitro*, albeit with lower efficiency than it inhibited SARS-CoV-1 [26].

Comparative genomic analysis reveals that several regions of the coronavirus genome are likely critical to virulence. The S1 domain of the spike protein, which contains the receptor binding motif, evolves more rapidly than *S*’s S2 domain [16,17]. However, even within the S1 domain, some regions are more conserved than others, with the receptors in S1’s N-terminal domain (S1-NTD) evolving more rapidly than those in its C-terminal domain (S1-CTD) [17]. Both S1-NTD and S1-CTD are involved in receptor binding and can function as RBDs to bind proteins and sugars [16], but RBDs in the S1-NTD typically bind to sugars, while those in the S1-CTD recognize protein receptors [5]. Viral receptors show higher affinity with protein receptors than sugar receptors [5], which suggests that positive selection on or relaxed conservation of the S1-NTD might reduce the risk of a deleterious mutation that would prevent binding. The SARS-CoV-2 S protein also contains an RRAR furin recognition site at the S1/S2 junction [9,23], setting it apart from both bat coronavirus RaTG13, with which it shares 96% genome sequence identity, and SARS-CoV-1 [136]. Such furin cleavage sites are commonly found in highly virulent influenza viruses, and as such may contribute to the heightened pathogenicity of SARS-CoV-2 [137,138]. The ongoing evolution of the spike protein can be seen from the genomic data. For example, the mutation D614G became dominant by the end of May 2020, soon after its initial appearance in mid-March [139,140], and a variant carrying two mutations (N501Y and 69–70del) that was first observed in the UK in October 2020 [141] has quickly spread around the world [142,143]. Effective cell entry is a critical component to pathogenesis and therefore an important process to understand when examining possible therapeutics.

## Mechanism of Transmission

Once a human host is infected with a virus, person-to-person viral transmission can occur through several possible mechanisms. The primary mechanisms associated with respiratory viruses are contact, droplet, and aerosol transmission [144]. Contact transmission can occur through either contact with a contagious person or contact with active viral particles on a contaminated surface [145]. This latter mode of transmission is also called fomite transmission [146]. Viral particles can enter the body if they then come in contact with the oral, nasal, eye, or other mucus membranes [145]. Droplet transmission occurs when a contagious individual sneezes, coughs, or exhales and produces respiratory droplets that can contain a large number of viral particles [145]. Contact with these droplets can occur through direct exposure to the droplets, such as breathing in droplets produced by a sneeze [145]. The droplets can also potentially settle on a surface and contribute to fomite transmission [145]. Aerosol transmission refers to much smaller particles (less than 5 micrometers) that are also produced by sneezing, coughing, or exhaling [144,145]. The small size of these particles allows them to remain suspended over a longer period of time and potentially to be moved by air currents [145]. Additionally, viral particles deposited on surfaces via large respiratory droplets can also later be aerosolized [145]. Droplet and/or contact transmission are both well-accepted modes of transmission for many viruses associated with common human illnesses, including influenza and rhinovirus [145]. The extent to which aerosol transmission contributes to the spread of respiratory viruses is less clear. In influenza A, for example, viral particles can be detected in aerosols produced by infected individuals, but the extent to which these particles drive the spread of influenza A infection remains under debate [144,145,147,148,149]. Regardless of its role in the spread of influenza A, however, aerosol transmission likely played a role in outbreaks such as the 1918 Spanish Influenza (H1N1) and 2009 “swine flu” (pH1N1) [149]. Contact, droplet, and aerosol transmission are therefore all worth evaluating when considering possible modes of transmission for a respiratory virus like SARS-CoV-2.

All three of these mechanisms have been identified as possible contributors to the transmission of HCoVs [145], including the highly pathogenic coronaviruses SARS-CoV-1 and MERS-CoV [150,151]. Transmission of SARS-CoV-1 is thought to proceed primarily through droplet transmission, but aerosol transmission is also considered possible [145], and fomite transmission may have also played an important role in some outbreaks [152]. Similarly, the primary mechanism of MERS transmission is thought to be droplets because inter-individual transmission appears to be associated with close interpersonal contact (e.g., household or healthcare settings), but aerosolized particles of the MERS virus have been reported to persist much more robustly than influenza A under a range of environmental conditions [153,154]. While droplet-based and contact transmission were initially put forward as the greatest concern for the spread of SARS-CoV-2 [155], as additional information has emerged, the possibility of aerosol transmission has also been raised [156,157,158]. For example, the detection of SARS-CoV-2 viral particles in air samples taken from hospitals treating COVID-19 patients led to the concern that the virus could be spreading via aerosols [159]. The stability of the virus both in aerosols and on a variety of surfaces appeared similar to that of SARS-CoV-1 [157]. However, while the possibility of aerosol transmission seems plausible, the evidence suggests that droplet transmission is the dominant mechanism driving the spread of the virus [160], and the risk of fomite transmission under real-world conditions is likely to be substantially lower than the conditions used for experimental analyses [161]. These mechanisms may differ in their relevance to different types of transmission events, such as transmission within households, nosocomial transmissions, and transmission in indoor versus outdoor spaces.

### Symptoms and Viral Spread

Other aspects of pathogenesis are also important to understanding how the virus spreads, especially the relationship between symptoms, viral shedding, and contagiousness. Symptoms associated with reported cases of COVID-19 range from mild to severe [1], but some individuals who contract COVID-19 remain asymptomatic throughout the duration of the illness [162]. The incubation period, or the time period between exposure and the onset of symptoms, has been estimated at five to eight days, with means of 4.91 (95% confidence interval (CI) 4.35–5.69) and 7.54 (95% CI 6.76–8.56) reported in two different Asian cities and a median of 5 (IQR 1 to 6) reported in a small number of patients in a Beijing hospital [163,164]. However, the exact relationship between contagiousness and viral shedding remains unclear. Estimates suggest that viral shedding can, in some cases, begin as early as 12.3 days (95% CI 5.9–17.0) before the onset of symptoms, although this was found to be very rare, with less than 0.1% of transmission events occurring 7 or more days before symptom onset [165]. Transmissibility appeared to peak around the onset of symptoms (95% CI −0.9 – 0.9 days), and only 44% (95% CI 30–57%) of transmission events were estimated to occur from presymptomatic contacts [165]. As these trends became apparent, concerns arose due to the potential for individuals who did not yet show symptoms to transmit the virus [166]. Recovered individuals may also be able to transmit the virus after their symptoms cease. Estimates of the communicable period based on twenty-four individuals who tested positive for SARS-CoV-2 prior to or without developing symptoms estimated that individuals may be contagious for one to twenty-one days, but they note that this estimate may be low [162]. In an early study, viral nucleic acids were reported to remain at observable levels in the respiratory specimens of recovering hospitalized COVID-19 patients for a median of 20 days and with a maximum observed duration through 37 days, when data collection for the study ceased [52]. As more estimates of the duration of viral shedding are released, they are beginning to converge around approximately three weeks from first positive PCR test and/or onset of symptoms (which, if present, are usually identified within three days of the initial PCR test). For example, in later studies, viral shedding was reported for up to 28 days following symptom onset [167] and for one to 24 days from first positive PCR test, with a median of 12 days [42]. On the other hand, almost 70% of patients were reported to still have symptoms at the time that viral shedding ceased, although all symptoms reduced in prevalence between onset and cessation of viral shedding [168]. The median time that elapsed between the onset of symptoms and cessation of viral RNA shedding was 23 days and between first positive PCR test and cessation of viral shedding was 17 days [168]. The fact that this study reported symptom onset to predate the first positive PCR test by an average of three days, however, suggests that there may be some methodological differences between it and related studies. Furthermore, an analysis of residents of a nursing home with a known SARS-CoV-2 case measured similar viral load in residents who were asymptomatic regardless of whether they later developed symptoms, and the load in the asymptomatic residents was comparable to that of residents who displayed either typical of atypical symptoms [169]. Taken together, these results suggest that the presence or absence of symptoms are not reliable predictors of viral shedding or of SARS-CoV-2 status (e.g, [170]). However, it should be noted that viral shedding is not necessarily a robust indicator of contagiousness. The risk of spreading the infection was low after ten days from the onset of symptoms, as viral load in sputum was found to be unlikely to pose a significant risk based on efforts to culture samples *in vitro* [167]. The relationship between symptoms, detectable levels of the virus, and risk of viral spread is therefore complex.

The extent to which asymptomatic or presymptomatic individuals are able to transmit SARS-CoV-2 has been a question of high scientific and community interest. Early reports (February and March 2020) described transmission from presymptomatic SARS-CoV-2-positive individuals to close family contacts [171,172]. One of these reports [172] also included a description of an individual who tested positive for SARS-CoV-2 but never developed symptoms. Later analyses also sought to estimate the proportion of infections that could be traced back to a presymptomatic or asymptomatic individual (e.g., [173]). Estimates of the proportion of individuals with asymptomatic infections have varied widely. The proportion of asymptomatic individuals on board the Diamond Princess cruise ship, which was the site of an early COVID-19 outbreak, was estimated at 17.9% [174]. In contrast, a model using the prevalence of antibodies among residents of Wuhan, China estimated a much higher rate of asymptomatic cases, at approximately 7 in 8, or 87.5% [175]. An analysis of the populations of care homes in London found that, among the residents (median age 85), the rate of asymptomatic infection was 43.8%, and among the caretakers (median age 47), the rate was 49.1% [176]. The duration of viral shedding may also be longer in individuals with asymptomatic cases of COVID-19 compared to those who do show symptoms [177]. As a result, the potential for individuals who do not know they have COVID-19 to spread the virus raises significant concerns. In Singapore and Tianjin, two cities studied to estimate incubation period, an estimated 40–50% and 60–80% of cases, respectively, were considered to be caused by contact with asymptomatic individuals [163]. An analysis of viral spread in the Italian town of Vo’, which was the site of an early COVID-19 outbreak, revealed that 42.5% of cases were asymptomatic and that the rate was similar across age groups [178]. The argument was thus made that the town’s lockdown was imperative for controlling the spread of COVID-19 because it isolated asymptomatic individuals. While more models are likely to emerge to better explore the effect of asymptomatic individuals on SARS-CoV-2 transmission, these results suggest that strategies for identifying and containing asymptomatic but contagious individuals are important for managing community spread.

### Estimating the Fatality Rate

Estimating the occurrence of asymptomatic and mild COVID-19 cases is important to identifying the mortality rate associated with COVID-19. The mortality rate of greatest interest would be the total number of fatalities as a fraction of the total number of people infected. One commonly reported metric is the case fatality rate (CFR), which compares the number of COVID-19 related deaths to the number of confirmed or suspected cases. However, in locations without universal testing protocols, it is impossible to identify all infected individuals because so many asymptomatic or mild cases go undetected. Therefore, a more informative metric is the infection fatality rate (IFR), which compares the known deaths to the estimated number of cases. It thus requires the same numerator as CFR, but divides by an approximation of the total number of cases rather than only the observed/suspected cases. IFR varies regionally, with some locations observed to have IFRs as low as 0.17% while others are as high as 1.7% [179]. Estimates of CFR at the national and continental level and IFR at the content level is maintained by the Centre for Evidence-Based Medicine [180]. Several meta-analyses have also sought to estimate IFR at the global scale. These estimates have varied; one peer-reviewed study aggregated data from 24 other studies and estimated IFR at 0.68% (95% CI 0.53%–0.82%), but a preprint that aggregated data from 139 countries calculated a global IFR of 1.04% (95% CI 0.77%−1.38%) when false negatives were considered in the model [179,181]. A similar prevalence estimate was identified through a repeated cross-sectional serosurvey conducted in New York City that estimated the IFR as 0.97% [182]. Examination of serosurvey-based estimates of IFR identified convergence on a global IFR estimate of 0.60% (95% CI 0.42%– 0.77%) [179]. All of these studies note that IFR varies widely by location, and it is also expected to vary with demographic and health-related variables such as age, sex, prevalence of comorbidities, and access to healthcare and testing [183]. Estimates of infection rates are becoming more feasible as more data becomes available for modeling and will be bolstered as serological testing becomes more common and more widely available.

## Dynamics of Transmission

Disease spread dynamics can be estimated using R_0_, the basic reproduction number, and R_t_, the effective reproduction number. Accurate estimates of both are crucial to understanding the dynamics of infection and to predicting the effects of different interventions. R_0_ is the average number of new (secondary) infections caused by one infected person, assuming a wholly susceptible population [184] and is one of the most important epidemiological parameters [185]. A simple mechanistic model used to describe infectious disease dynamics is a susceptible-infected-recovered compartmental model [186,187]. In this model, individuals move through three states: susceptible, infected, and recovered; two parameters, and *γ* and *β*, specify the rate at which the infectious recover, and the infection transmission rate, respectively, and R_0_ is estimated as the ratio of *β* and *γ* [185,188]. A pathogen can invade a susceptible population only if R_0_ > 1 [185,189]. The spread of an infectious disease at a particular time t can be quantified by R_t_, the effective reproduction number, which assumes that part of the population has already recovered (and thus gained immunity to reinfection) or that mitigating interventions have been put into place. For example, if only a fraction S_t_ of the population is still susceptible, R t = S_t_ × R_0_. When R_t_ is greater than 1, an epidemic grows (i.e., the proportion of the population that is infectious increases); when R_t_ is less than 1, the proportion of the population that is infectious decreases. R_0_ and R_t_ can be estimated directly from epidemiological data or inferred using susceptible-infected-recovered-type models. To accurately capture the dynamics of SARS-CoV-2, the addition of a fourth compartment, i.e. a susceptible-exposed-infectious-recovered model may be appropriate.

Estimates of R_0_ for COVID-19 lie in the range R_0_=1.4–6.5 [190,191,192]. Variation in R_0_ is expected between different populations, and the estimated values of R_0_ discussed below are for specific populations in specific environments. The different estimates of R_0_ should not necessarily be interpreted as a range of estimates of the same underlying parameter. In one study of international cases, the predicted value was R_0_=1.7 [193]. In China (both Hubei province and nationwide), the value was predicted to lie in the range R_0_=2.0–3.6 [190,194,195]. Another estimate based on a cruise ship where an outbreak occurred predicted R_0_=2.28 [196]. Susceptible-exposed-infectious-recovered model-derived estimates of R_0_ range from 2.0 – 6.5 in China [197,198,199,200] to R_0_=4.8 in France [201]. Using the same model as for the French population, a study estimated R_0_=2.6 in South Korea [201], which is consistent with other studies [202]. From a meta-analysis of studies estimating R_0_, [191] the median R_0_ was estimated to be 2.79 (IQR 1.16) based on twelve studies published between January 1 and February 7, 2020.

Inference of the effective reproduction number can provide insight into how populations respond to an infection and the effectiveness of interventions. In China, R_t_ was predicted to lie in the range 1.6–2.6 in January 2020, before travel restrictions [203]. R_t_ decreased from 2.35 one week before travel restrictions were imposed (January 23, 2020), to 1.05 one week after. Using their model, the authors also estimated the probability of new outbreaks occurring. Assuming individual-level variation in transmission comparable to that of MERS or SARS, the probability of a single individual exporting the virus and causing a large outbreak is 17–25%, and assuming variation like that of SARS and transmission patterns like those observed for COVID-19 in Wuhan, the probability of a large outbreak occurring after ≥4 infections exist at a new location is greater than 50%. An independent study came to similar conclusions, finding R_t_=2.38 in the two-week period before January 23 with a decrease to R_t_ = 1.34 (using data from January 24 to February 3) or R_t_=0.98 (using data from January 24 to February 8) [192]. In South Korea, R_t_ was inferred for February through March 2020 in two cities, Daegu (the center of the outbreak) and Seoul [202]. Metro data was also analyzed to estimate the effects of social distancing measures. R_t_ decreased in Daegu from around 3 to <1 over the period that social distancing measures were introduced. In Seoul, R_t_ decreased slightly, but remained close to 1 (and larger than R_t_ in Daegu). These findings indicate that social distancing measures appeared to be effective in containing the infection in Daegu, but in Seoul, R_t_ remained above 1, meaning secondary outbreaks remained possible. The study also shows the importance of region-specific analysis: the large decline in case load nationwide was mainly due to the Daegu region and could mask persistence of the epidemic in other regions, such as Seoul and Gyeonggi-do. In Iran, estimates of R_t_ declined from 4.86 in the first week to 2.1 by the fourth week after the first cases were reported [204]. In Europe, analysis of 11 countries inferred the dynamics of R_t_ over a time range from the beginning of the outbreak until March 28, 2020, by which point most countries had implemented major interventions (such as stay-at-home orders, public gathering bans, and school closures) [205]. Across all countries, the mean R_t_ before interventions began was estimated as 3.87; R_t_ varied considerably, from below 3 in Norway to above 4.5 in Spain. After interventions, R_t_ decreased by an average of 64% across all countries, with mean R_t_=1.43. The lowest predicted value was 0.97 for Norway and the highest was 2.64 for Sweden, which could be related to the fact that Sweden did not implement social distancing measures on the same scale as other countries. The study concludes that while large changes in R_t_ are observed, it is too early to tell whether the interventions put into place are sufficient to decrease R_t_ below 1.

More generally, population-level epidemic dynamics can be both observed and modeled [188]. Data and empirically determined biological mechanisms inform models, while models can be used to try to understand data and systems of interest or to make predictions about possible future dynamics, such as the estimation of capacity needs [206] or the comparison of predicted outcomes among prevention and control strategies [207,208]. Many current efforts to model R_t_ have also led to tools that assist the visualization of estimates in real time or over recent intervals [209,210]. These are valuable resources, yet it is also important to note that the estimates arise from models containing many assumptions and are dependent on the quality of the data they use, which varies widely by region.

## Molecular Signatures and Transmission

Genetic variation in SARS-CoV-2 has been used to elucidate patterns over time and space. Mutations observed in individual SARS-CoV-2 genome sequences can be used to trace transmission patterns and have provided insights during outbreak investigations [211,212,213]. Similar mutations observed in several patients may indicate that the patients belong to the same transmission group. The tracking of SARS-CoV-2 mutations is recognized as an essential tool for controlling future outbreaks and tracing the path of the spread of SARS-CoV-2. Efforts vary widely by country: the UK has coordinated has coordinated a national database of viral genomes [214]; no such coordination has been achieved in the USA. Several studies used phylogenetic analysis to determine the source of local COVID-19 outbreaks in Connecticut (USA), [215], the New York City area (USA) [216], and Iceland [217]. There is an ongoing effort to collect SARS-CoV-2 genomes throughout the COVID-19 outbreak, and as of January 18, 2021 more than 381,000 genome sequences have been collected from patients. The sequencing data can be found at GISAID [218], NCBI [219], and COVID-19 data portal [220].

## Conclusions

The novel coronavirus SARS-CoV-2 is the third HCoV to emerge in the 21st century, and research into previous HCoVs has provided a strong foundation for characterizing the pathogenesis and transmission of SARS-CoV-2. Critical insights into how the virus interacts with human cells have been gained from previous research into HCoVs and other viral infections. As with other HCoVs, the immune response to SARS-CoV-2 is likely driven by detection of its spike protein, which allows it to enter cells through ACE2. Epithelial cells have also emerged as the major cellular target of the virus, contextualizing the respiratory and gastrointestinal symptoms that are frequently observed in COVID-19. Many of the mechanisms that facilitate the pathogenesis of SARS-CoV-2 are currently under consideration as possible targets for the treatment or prevention of COVID-19. Research in other viruses also provides a foundation for understanding the transmission of SARS-CoV-2 among people and can therefore inform efforts to control the virus’s spread. The extent to which aerosol and fomite transmission contribute to the spread of SARS-CoV-2 remains a question: in general, much like SARS-CoV-1 and MERS-CoV, this virus seems to be spread primarily by droplet transmission. Asymptomatic transmission was also a concern in the SARS outbreak of 2002–03 and, as in current pandemic, presented challenges for estimating rates of infection [221]. However, in the current pandemic, we have been fortunate to be able to build on top of 18 years of SARS-CoV-1 research in order to rapidly ascertain the identity and behavior of the virus.

Even with the background obtained from research in SARS and MERS, COVID-19 has revealed itself to be a complex and difficult-to-characterize disease that has many possible presentations that vary with age. Variability in presentation, including cases with no respiratory symptoms or with no symptoms altogether, were also reported during the SARS epidemic at the beginning of the 21st century [221]. The variability of both which symptoms present and their severity have presented challenges for public health agencies seeking to provide clear recommendations regarding which symptoms indicate SARS-CoV-2 infection and should prompt isolation. Asymptomatic cases add complexity both to efforts to estimate statistics such as R_0_ and R_t_, which are critical to understanding the transmission of the virus, and IFR, which is an important component of understanding its impact on a given population. The development of diagnostic technologies over the course of the pandemic has facilitated more accurate identification, including of asymptomatic cases. As more cases have been diagnosed, the health conditions and patient characteristics associated with more severe infection have also become more clear, although there are likely to be significant sociocultural elements that also influence these outcomes. While many efforts have focused on adults, and especially older adults because of the susceptibility of this demographic, additional research is needed to understand the presentation of COVID-19 and MIS-C in pediatric patients. As more information is uncovered about the pathogenesis of HCoV and SARS-CoV-2 specifically, the diverse symptomatology of COVID-19 has and likely will continue to conform with the ever-broadening understanding of how SARS-CoV-2 functions within a human host.

While the SARS-CoV-2 virus is very similar to other HCoV in several ways, including in its genomic structure and the structure of the virus itself, there are also some differences that may account for differences in the COVID-19 pandemic compared to the SARS and MERS epidemics of the past two decades. The R_0_ of SARS-CoV-2 has been estimated to be similar to SARS-CoV-1 but much higher than that of MERS-CoV [222,222]. While the structures of the viruses are very similar, evolution among these species may account for differences in their transmissibility and virulence. For example, the acquisition of a furin cleavage site the S1/S2 boundary within the SARS-CoV-2 S protein may be associated with increased virulence. Additionally, concerns have been raised about the accumulation of mutations within the SARS-CoV-2 species itself, and whether these could influence virulence. The coming of age of genomic technologies has made these types of analyses feasible, and genomics research characterizing changes in SARS-CoV-2 along with temporal and spatial movement is likely to provide additional insights into whether within-species evolution influences the effect of the virus on the human host. Additionally, the rapid development of sequencing technologies over the past decade has made it possible to rapidly characterize the host response to the virus. For example, proteomics analysis of patient-derived cells revealed candidate genes whose regulation is altered by SARS-CoV-2 infection, suggesting possible approaches for pharmaceutical invention and providing insight into which systems are likely to be disrupted in COVID-19 [127]. As more patient data becomes available, the biotechnological advances of the 2000s are expected to allow for more rapid identification of potential drug targets than was feasible during the SARS, or even MERS, pandemic.

Thus, though the COVID-19 crisis is still evolving, the insights acquired over the past 20 years of HCoV research have provided a solid foundation for understanding the SARS-CoV-2 virus and the disease it causes. As the scientific community continues to respond to COVID-19 and to elucidate more of the relationships between viral pathogenesis, transmission, and symptomatology, and as more data about the regulatory shifts associated with COVID-19 become available, this understanding will no doubt continue to evolve and to reveal additional connections among virology, pathogenesis, and health. As additional information becomes available, this review will be updated to reflect the changing state of research in this area. At present, understanding the SARS-CoV-2 virus and its pathogenesis is critical to a holistic understanding of the COVID-19 pandemic.

## Importance

COVID-19 involves a number of organ systems and can present with a wide range of symptoms. Understanding how the virus infects epithelial cells, however, serves to contextualize how these systems connect. Similarly, while the modes of viral transmission have been under debate throughout much of 2020 and the beginning of 2021, the available research suggests that these patterns are very similar to those seen in the closely related viruses SARS-CoV-1 and possibly MERS-CoV. Exploring the structure, phylogeny, and pathogenesis of the virus therefore helps to guide interpretation of the broader impacts of the virus on the human body and on human populations. For this reason, an in-depth exploration of viral mechanisms is critical to a robust understanding of the COVID-19 pandemic.
